# Cardiac MR-derived indices are stronger predictors of resource use and risk than jugular venous pressure, in paediatric patients with functionally single ventricles, prior to completion of total cavopulmonary connection (TCPC)

**DOI:** 10.1186/1532-429X-17-S1-O56

**Published:** 2015-02-03

**Authors:** Marina Hughes, Sylvia Krupickova, Troy Dominguez, Michael Broadhead, Oliver Tann, Angus McEwan, Vivek Muthurangu, Andrew Taylor

**Affiliations:** 1 Great Ormond Street Hospital for Children NHS Foundation Trust, London, UK; 2 University College London, London, UK

## Background

Cardiac MR (CMR) imaging contributes to crucial anatomic and physiologic data for paediatric patients with functionally single ventricles, prior to total cavopulmonary connection (TCPC), and may obviate isolated pressure data for predicting risk and outcome in these complex patients.

## Methods

Outcome data for all patients undergoing inter-stage pre-TCPC CMR between 2007-2014 was analysed. Pre-determined outcome measures included survival, duration of peri-TCPC hospitalisation and TCPC fenestration. MRI was performed under general anaesthetic with simultaneous transduction of jugular venous pressure (JVP), as per our Unit protocol.

CMR-derived data included volumes and function of single ventricle, through-plane phase contrast flow volumes, contrast-enhanced angiography and 3D SSFP images. Flow was measured in ascending aorta, SVC, IVC, pulmonary arteries and pulmonary veins bilaterally. Thus systemic to pulmonary (S-P) collateral flow, net PA flow and SVC:IVC flow was quantified. 3D images allowed quantitative scoring of systemic veins off-loading the SVC system into IVC : 0= no SVC-IVC offloading veins seen, 1= few, small, low calibre veins, 2= large, obvious veins.

## Results

A total of 100 patients had pre-TCPC CMR as per default inter-stage imaging protocol. The only patients excluded from this analysis, not undergoing CMR, underwent extra-protocol cardiac CT or interventional cardiac catheterisation.

Of the 100 children undergoing CMR, the median (range) follow-up period was 4.2 (0.3-9.4) years. 97 patients have been successfully converted to TCPC, 2 were deemed not suitable for TCPC, 1 awaits surgery. 4 patients have died following TCPC.

Median (range) age at CMR was 3.4 (1.5-10.1) years, and weight 14.4 (9.4-40) kg.

JVP measured at CMR did not relate to survival, nor duration of peri-TCPC hospital stay. JVP did not reflect ventricular dominance, ejection fraction, arch or branch PA stenosis, or S-P collateral flow. JVP did correlate inversely with ratio SVC:IVC flow volume (r = -0.24, p=0.04).

Conversely, CMR-derived indices, including: PA obstruction or hypoplasia, S-P collateral flow, the presence of obvious SVC-IVC offloading veins (Score 2), and a surrogate of pulmonary vascular resistance, (JVP/net PA flow) significantly related to decreased survival and correlated significantly with increased length of hospital stay.

## Conclusions

For inter-stage patients with BCPC, the instantaneous jugular venous pressure is confounded by ventilatory and volume factors, and venous offloading into pulmonary or IVC circulation. In our cohort of TCPC patients examined with CMR, we have shown that CMR-derived factors are stronger predictors of resource use and risk than isolated jugular venous pressure measurement.

## Funding

No authors have received specific funding for this study.

**Table 1:** Descriptive variables for the study cohort - all patients undergoing pre-TCPC CMR under general anaesthetic 2007 - 2014 (n = 100).

VARIABLE	MEDIAN (Range) or FRACTION
Total cohort	100

Male	63/100

Age at BCPC (months)	6.9 (1.2 - 80.3)

Age at CMR (months)	40.4 (17.7 - 121.2)

Weight at CMR (kg)	14.5 (9.4 - 40)

Age at TCPC (years)	3.9 (2.1 - 10.5)

Follow-up period from CMR (years)	4.2 (0.3 - 9.4)

Dominant RV : Dominant LV	55/100

Arch reconstruction or DKS	54 / 100

Branch PA stenosis or hypoplasia	60/100

Number with native PA forward flow	17/100

Jugular venous pressure at CMR (mmHg)	12 (5 - 20)

ntricular ejection fraction (%)	56 (36 - 77)

A-V valve regurgitant fraction (%)	5 (0 - 25)

Net PA flow (L/min)	1.3 (0.6 - 3.6)

Net Ao flow/BSA (Cardiac index) (L/min/m2)	4.4 (2.5 - 7.6)

SVC / IVC flow ratio	1.36 (0.50 - 2.21)

Proportion collateral flow of pulm venous return (%)	35 (0 - 68)

Number with obvious SVC offloading veins (Score = 2)	33 / 100

Peri-TCPC hospital length of stay (days)	12 (4 - 69)

Death following TCPC	4 / 100

**Figure 1:**
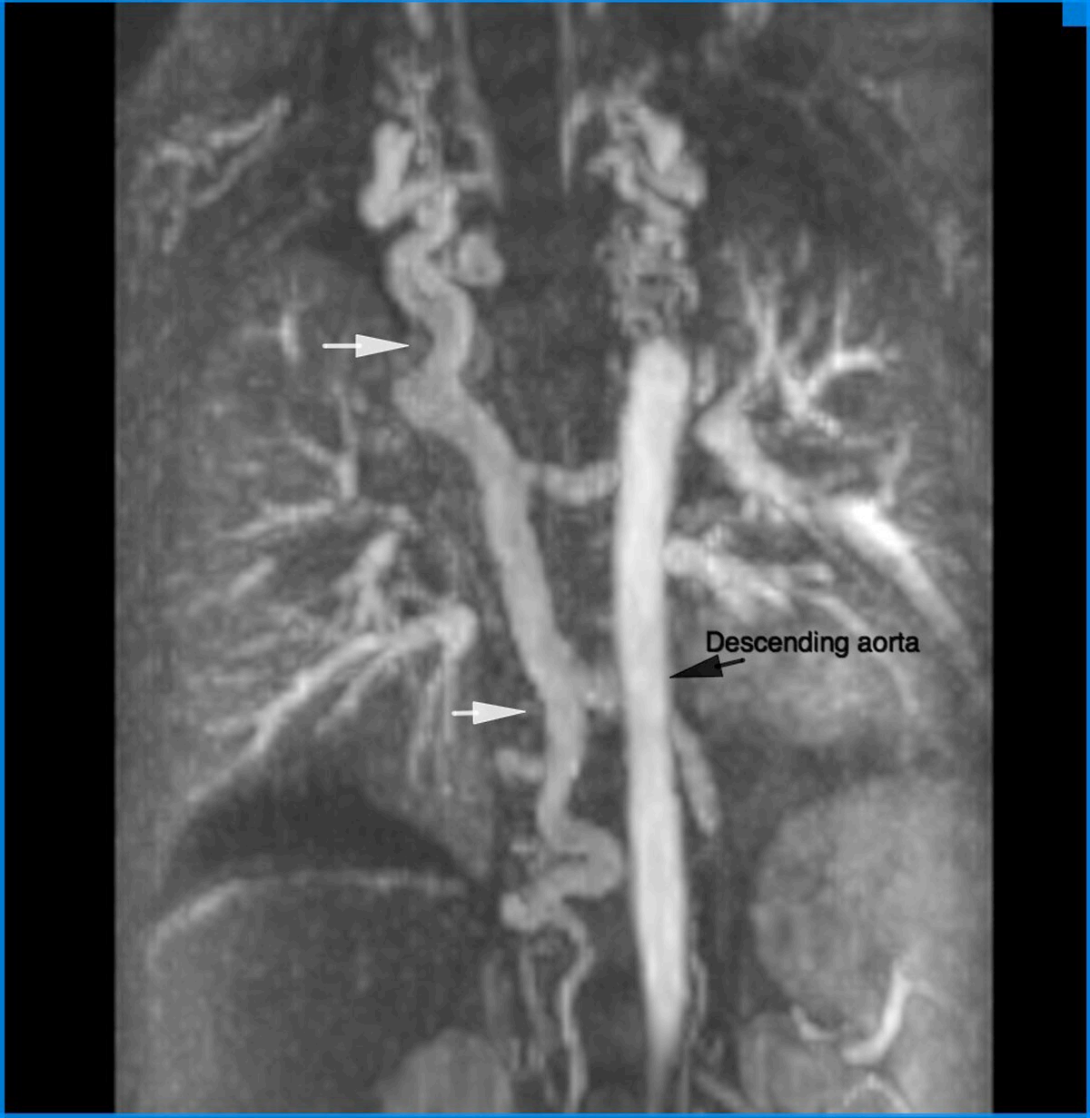
MPR reconstruction in the coronal plane from an angiographic image, showing a large, tortuous SVC-IVC offloading vein, which appears to have recannalated the azygous pathway

